# Predicting miRNA-disease associations based on multi-view information fusion

**DOI:** 10.3389/fgene.2022.979815

**Published:** 2022-09-27

**Authors:** Xuping Xie, Yan Wang, Nan Sheng, Shuangquan Zhang, Yangkun Cao, Yuan Fu

**Affiliations:** ^1^ Key Laboratory of Symbol Computation and Knowledge Engineering of Ministry of Education, College of Computer Science and Technology, Jilin University, Changchun, China; ^2^ School of Artificial Intelligence, Jilin University, Changchun, China; ^3^ Institute of Biological, Environmental and Rural Sciences, Aberystwyth University, Aberystwyth, United Kingdom

**Keywords:** miRNA-disease associations, multi-view, deep learning, graph convolutional networks, convolutional neural networks

## Abstract

MicroRNAs (miRNAs) play an important role in various biological processes and their abnormal expression could lead to the occurrence of diseases. Exploring the potential relationships between miRNAs and diseases can contribute to the diagnosis and treatment of complex diseases. The increasing databases storing miRNA and disease information provide opportunities to develop computational methods for discovering unobserved disease-related miRNAs, but there are still some challenges in how to effectively learn and fuse information from multi-source data. In this study, we propose a multi-view information fusion based method for miRNA-disease association (MDA)prediction, named MVIFMDA. Firstly, multiple heterogeneous networks are constructed by combining the known MDAs and different similarities of miRNAs and diseases based on multi-source information. Secondly, the topology features of miRNAs and diseases are obtained by using the graph convolutional network to each heterogeneous network view, respectively. Moreover, we design the attention strategy at the topology representation level to adaptively fuse representations including different structural information. Meanwhile, we learn the attribute representations of miRNAs and diseases from their similarity attribute views with convolutional neural networks, respectively. Finally, the complicated associations between miRNAs and diseases are reconstructed by applying a bilinear decoder to the combined features, which combine topology and attribute representations. Experimental results on the public dataset demonstrate that our proposed model consistently outperforms baseline methods. The case studies further show the ability of the MVIFMDA model for inferring underlying associations between miRNAs and diseases.

## 1 Introduction

MicroRNAs (miRNAs) are endogenous non-coding RNAs of approximately 21–23 nucleotides that play an important role in the regulation of gene expression (Bartel, 2004). A large number of studies have shown that miRNAs are involved in various biological processes, including metabolism, cell proliferation, cell cycle regulation, and differentiation (Cheng et al., 2005; Miska, 2005; Carleton et al., 2007; Bartel, 2009), and abnormal expression of miRNAs is related to the pathogenesis of various diseases such as cancer (Calin and Croce, 2006; Small and Olson, 2011; Bracken et al., 2016; Metzinger-Le Meuth and Metzinger, 2019; Sereshgi et al., 2019). Considering the important roles of miRNAs in different diseases, the identification of potential associations between miRNAs and diseases is helpful for the understanding of disease pathogenesis and the diagnosis, treatment and prognosis of diseases. Traditional biological assay methods for discovering disease-related miRNAs are time-consuming and expensive. Therefore, with the accumulation of biological data and the improvement of computational power, more and more researchers propose to predict potential miRNA-disease associations (MDAs) by using computational methods.

Based on the hypothesis that miRNAs with similar functions are more likely to be associated with diseases with similar phenotypes and vice versa (Bandyopadhyay et al., 2010), many computational models have been developed to predict MDAs. For example, Jiang et al. (2010) used a hypergeometric distribution model to evaluate the probability scores of unknown MDAs based on a phenome-microRNAome network. But this model only considers the direct neighbor information of each node and ignores the indirect neighbors. Subsequently, Xuan et al. (2013) developed a computational model HDMP based on k most similar neighbors to infer disease-related miRNAs. To improve the prediction result, HDMP puts forward to estimate miRNA functional similarity by integrating the information content of disease terms and phenotypic similarity between diseases. However, HDMP only considers the local information of the network and is not suitable for predicting potential miRNAs for novel diseases without known related miRNAs. Therefore, some methods that consider global network information have been proposed by some researchers. To make good use of structural information, Chen et al. (2012) used the random walk with restart on the miRNA functional similarity network to infer the potential associations between miRNAs and diseases. The algorithm still has the limitation that it is not applicable to new diseases and new miRNAs. Although researchers have proposed many new models (Chen et al., 2016; You et al., 2017; Chen et al., 2018b) to solve this problem, the above similarity-based methods still cannot effectively capture the complex relationships of miRNA-disease pairs.

In addition, matrix completion-based methods are also often used for biomedical link prediction due to their ability to explore the intrinsic and shared structures of heterogeneous data sources (Ou-Yang et al., 2022). Specifically, we predict potential connections by filling in the missing entries of part of the observed matrix when using this method for MDA inference. Li et al. (2017) put forward an efficient matrix completion model to infer novel MDAs, called MCMDA. Subsequently, to solve the problem that MCMDA cannot be used for new diseases, Chen et al. (2018a) designed a new computational model based on inductive matrix completion to predict potential miRNAs associated with diseases, which uses integrated miRNA similarity, disease similarity and validated MDA pairs to complement missing MDAs. Meanwhile, Xiao et al. (2018) presented a graph regularized non-negative matrix factorization method to take full advantage of the intrinsic geometric structure of the data, which enables it to effectively discover potential relationships between miRNAs and diseases, including new diseases and new miRNAs. Some matrix completion-based methods have been developed to infer underlying associations between miRNAs and diseases (Gao et al., 2020; Zhang et al., 2020; Chen et al., 2021).

In recent years, as machine learning methods have been widely used in various fields, some machine learning-based models have been presented to further improve the prediction performance of miRNA-disease potential associations. For example, Xu et al. (2011) calculated four topological features of miRNAs and constructed a support vector machine classifier to reveal the relationships between diseases and miRNAs. Since samples are randomly selected from unknown miRNA-disease relationship pairs as negative samples, these negative samples are unreliable, and they may be positive samples that have not been experimentally verified. Given the limitations of existing methods, Chen and Yan (2014) used a semi-supervised learning-based computational model of regularized least squares to identify miRNAs that may be associated with diseases. This method can be used for diseases without validated relevant miRNAs and avoid the selection of negative samples by using semi-supervision. However, with the rapid growth of biomedical data, traditional machine learning methods are not suitable for complex and changeable data, while deep learning has shown good performance in utilizing unstructured data (Sheng et al., 2022; Zhang et al., 2022). Peng et al. (2019) used auto-encoder to reduce the dimensionality of features and calculated miRNA-disease relationship scores by the convolutional neural network (CNN). Li et al. (2020) proposed to use latent feature representations of miRNAs and diseases, respectively learned by graph convolutional networks (GCNs) (Kipf and Welling, 2016), as input for neural inductive matrix completion to obtain scores for unknown miRNA-disease pairs. Tang et al. (2021) presented a multi-view multichannel attention graph convolutional network (MMGCN) to identify new disease-related miRNAs, which uses GCNs to learn the embeddings of miRNAs and diseases, furthermore adopts multi-channel attention to enhance the learned latent representations. The GNN based on link representation proposed by Kang et al. (2022) employed the GCN to obtain node embeddings and then obtained the improved intermolecular relationship scores according to the designed propagation rule and layer-wise fusing rule. Although there are some methods using deep learning for MDA prediction, many of them ignore the effective learning and fusion of information from different data sources, such as some methods simply utilize one type information, some simply fill in the missing values of one type of information with other types of information, and some ignore information in known associations. Thus, the methods that better utilize multi-source data information to identify underlying disease-related miRNAs should be further explored.

In this study, we present a novel MDA prediction method based on multi-view information fusion (MVIFMDA), which attempts to effectively preserve the topological and attribute information from multi-source data. The basic idea of MVIFMDA is as follows. We firstly use multi-source data to construct the known association network of miRNA-disease and the similarity networks of miRNA and disease, including miRNA sequence similarity network, miRNA functional similarity network, disease semantic similarity network, and disease functional similarity network. And then multiple heterogeneous networks between miRNAs and diseases are constructed based on the association network and similarity networks of miRNA and disease. Secondly, GCNs are employed to learn various topological representations of miRNAs and diseases according to different heterogeneous network views, respectively. Furthermore, the attention strategy at the topology representation level is established to obtain more informative topology embeddings by effectively learning the importance of different topology features. Meanwhile, CNNs are adopted to respectively get the attribute representations of the miRNAs and diseases based on the various miRNA similarity and disease similarity views. Finally, the combined miRNA and disease embeddings are fed into a bilinear decoder to calculate the MDA scores. 5-Fold cross-validation (5-CV) and case studies demonstrate that the MVIFMDA model extracts more information from multiple biological data sources and is suitable for MDA prediction.

## 2 Materials and methods

**FIGURE 1:**
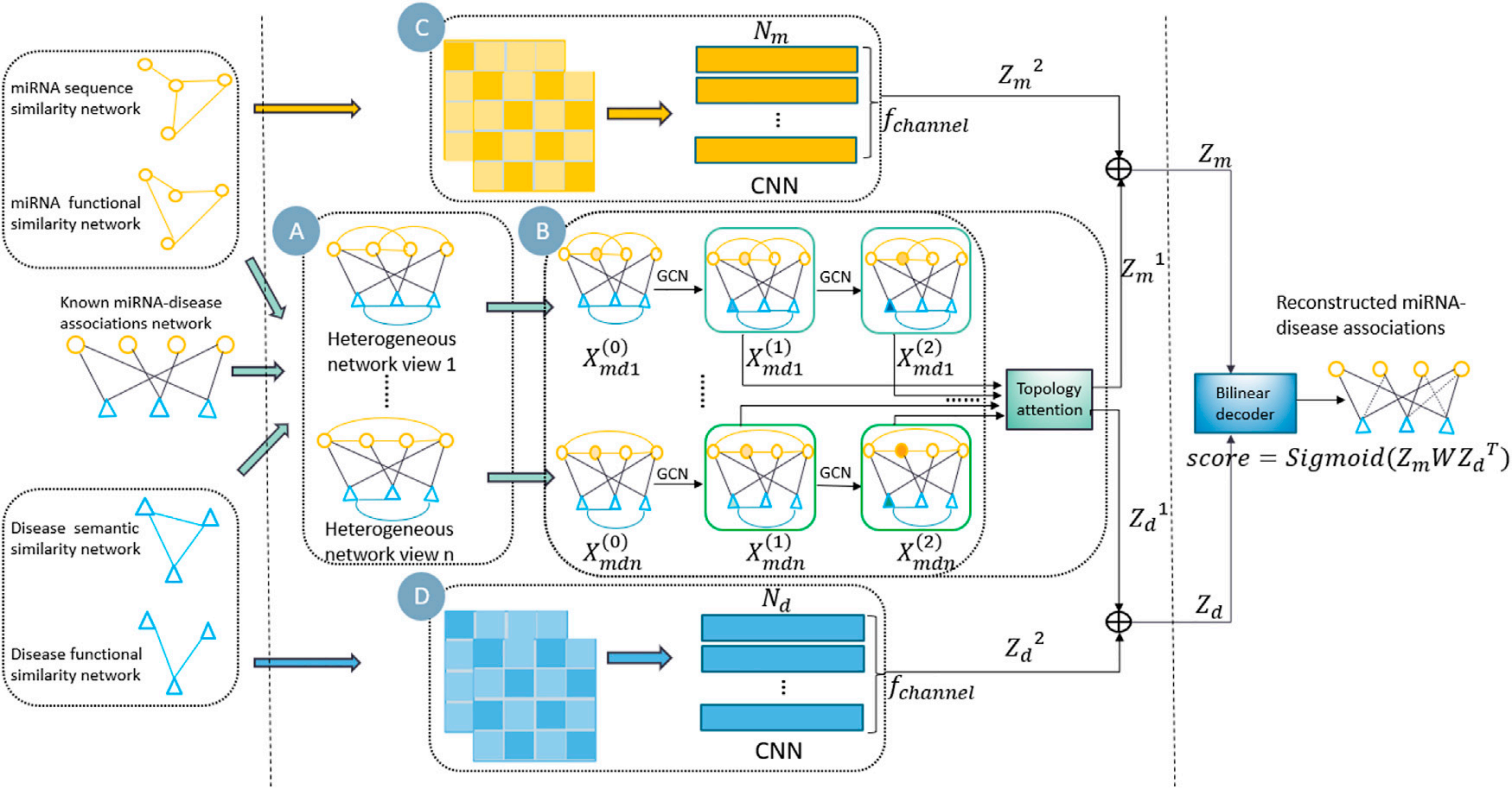
Overview of MVIFMDA. **(A)** Construction of multiple miRNA-disease heterogeneous networks using known MDAs and the similarities of miRNAs and diseases. **(B)** Encoding of heterogeneous network views by GCN to extract topology representations, and using topology representation level attention mechanism to adaptively fuse the different topology information. **(C)** and **(D)** Encoding of similarity views by CNN to obtain attribute representations of miRNAs and diseases, respectively.

In this study, we propose a new multi-view information fusion model named MVIFMDA for MDA prediction. The framework of MVIFMDA is shown in Figure 1. We firstly construct miRNA-disease heterogeneous networks based on known associations and various similarities of miRNA and disease (Figure 1A). Additionally, GCNs are adopted to encode heterogeneous network views including different information, and an attention mechanism is designed to adaptively integrate different topology representations for miRNAs and diseases obtained from GCNs (Figure 1B). Meanwhile, the attribute representations of the miRNAs and diseases are learned by utilizing the CNN encoder (Figures 1C,D). Finally, the bilinear decoder combines the topology and attribute representations of miRNAs and diseases to predict the association scores between miRNAs and diseases.

### 2.1 Dataset

We downloaded experimentally validated human miRNA-disease relationships from HMDD v3.2 (Huang et al., 2019), which is a curated database. To take full advantage of multiple biomedical data, we obtained the medical subject headings (MeSH) descriptors from the National Library of Medicine (https://www.nlm.nih.gov/), which provides semantic information of diseases through directed acyclic graphs (DAGs). And disease-gene associations and weighted gene-gene relationships were obtained from DisGeNET (Piñero et al., 2020) and HumanNet (Hwang et al., 2019), respectively. In addition, we downloaded the sequence information of miRNAs from miRbase (Kozomara et al., 2019), and the miRNA-gene relationships were got from miRTarBase (Huang et al., 2020). By taking the intersection of data from multiple data sources and merging duplicate data, we finally acquired 12,446 associations among 853 miRNAs and 591 diseases for the next association prediction, furthermore used these processed data to calculate the similarities of miRNAs and diseases and build heterogeneous networks between miRNAs and diseases.

### 2.2 Construction of heterogeneous networks

#### 2.2.1 Human miRNA-disease associations

We employ the obtained known human relationship pairs between miRNAs and diseases to construct the association matrix $A\in R^{N_{m}\times N_{d}}$ , where $N_{m}$ and $N_{d}$ represent the number of miRNAs and diseases, respectively. When there is an observed association between miRNA i and disease j , $A_{ij}=1$ , that is, there is an edge with the weight of 1 between the miRNA i node and the disease j node. When there is an unknown or unobserved association between miRNA i and disease j, $A_{ij}=0$ , which means that there is no edge between the two nodes.

#### 2.2.2 Disease semantic similarity

As shown in (Wang et al., 2010a), DAGs can be used to compute the semantic similarity of diseases. For example, a disease d can be represented as $DAG\left(d\right)=\left(d,T_{d},E_{d}\right)$ , where $T_{d}$ is the disease set composed of all ancestor nodes of disease d (including itself), and $E_{d}$ denotes all direct connections between nodes. In DAG(d) , the semantic contribution of disease t to disease d is defined as follows:

$$\left\{ \begin{array}{c} D_{d}\left(t\right)=1,\;if\;t=d\\ D_{d}\left(t\right)=\max\left\{∆*D_{d}\left(t^{\prime }\right)\;|\;t^{\prime }\in children\;of\;t\right\},\;if\;t\neq d \end{array}\right.\;\;,$$ (1)

where the semantic contribution factor ∆ is set to 0.5 (Wang et al., 2010b), which means that the farther the disease is from the disease d , the smaller the semantic contribution to the disease d . Then, the semantic similarity performance between disease i and disease j is calculated as follows:

$${SD}^{1}\left(i,j\right)=\frac{\sum_{t\in T\left(i\right)\cap T\left(j\right)}\left(D_{i}\left(t\right)+D_{j}\left(t\right)\right)}{\sum_{t\in T\left(i\right)}D_{i}\left(t\right)+\sum_{t\in T\left(j\right)}D_{j}\left(t\right)}\;.$$ (2)

According to Eq. 2, we can finally get the weight matrix of the disease semantic similarity network ${SD}^{1}\in R^{N_{d}\times N_{d}}$ , where if ${{SD}^{1}}_{ij}\neq 0$ , it means that there is an edge with a weight of ${{SD}^{1}}_{ij}$ between the node i and j . On the contrary, when ${{SD}^{1}}_{ij}=0$ , it indicates that there is no semantically similar edge between the node i and j .

#### 2.2.3 Disease functional similarity

Driven by the hypothesis that similar disease tendencies interact with similar genes (Xu and Li, 2006; Wei and Liu, 2020), we calculate the functional similarity of diseases utilizing the relationship between disease and gene. The gene functional interaction network can be obtained from HumanNet, where it provides a log-likelihood score (LLS) for each gene interaction to assess the probability of functional connectivity between genes (Lee et al., 2011; Hwang et al., 2019). We obtain the similarity ${LLS}_{N}$ between genes through min-max normalization based on LLS, and then the similarity score between a gene g and a set of genes $GS=\left\{g_{1},g_{2},\cdots ,g_{k}\right\}$ is defined as follows:

$$S\left(g,GS\right)=\max_{g_{i}\in GS}\left(S\left(g,g_{i}\right)\right)\;,$$ (3)

where $S\left(g,g_{i}\right)$ represents the functional similarity score between gene g and gene $g_{i}$ . It is defined as follows:

$$S\left(g,g_{i}\right)=\left\{ \begin{array}{c} \;1\;,\;if\;g=g_{i}\\ {LLS\;}_{N}\left(g,g_{i}\right),\;if\;g\neq g_{i} \end{array}\right.\;,$$ (4)

where ${LLS\;}_{N}\left(g,g_{i}\right)=0$ when there is no linkage between the genes g and $g_{i}$ . Finally, we get the functional similarity between disease i and disease j as follows:

$${SD}^{2}\left(i,j\right)=\frac{\sum_{g\in {GS}_{i}}S\left(g,{GS}_{j}\right)+\sum_{g\in {GS}_{j}}S\left(g,{GS}_{i}\right)}{\left|{GS}_{i}\right|+\left|{GS}_{j}\right|}\;,$$ (5)

where ${GS}_{i}$ and ${GS}_{j}$ denote gene sets related to diseases i and j respectively, and $\left|{GS}_{i}\right|$ and $\left|{GS}_{j}\right|$ are the cardinality of the gene sets. Analogously, according to Eq. 5, we finally get the weight matrix of the disease functional similarity network ${SD}^{2}\in R^{N_{d}\times N_{d}}$ .

#### 2.2.4 miRNA sequence similarity

To measure the similarity of miRNA sequences, we employ the Needleman-Wunsch Algorithm (Needleman and Wunsch, 1970) to quantify the similarity between two miRNAs by sequence alignment. In addition, we normalize the sequence similarity score Score(i,j) between miRNA i and miRNA j to the range of [0,1] by min-max normalization, which can be written as follows:

$${SM}^{1}\left(i,j\right)=\frac{Score\left(i,j\right)-{Score}_{\min}}{{Score}_{\max}-{Score}_{\min}}\;,$$ (6)

where ${Score}_{\min}$ and ${Score}_{\max}$ represent the minimum and maximum similarity scores among all miRNA sequence pairs, respectively. Similar to the disease similarity network, we obtain a miRNA sequence similarity network with the edge weight matrix ${SM}^{1}\in R^{N_{m}\times N_{m}}$ .

#### 2.2.5 miRNA functional similarity

Similar to the calculation of disease functional similarity, we utilize the relationships between miRNAs and genes to calculate miRNA functional similarity, which avoids the dependence on known associations between miRNAs and diseases and enables the similarity calculation of new miRNAs (Xiao et al., 2018; Xiao et al., 2021). Analogously, we can define the functional similarity between miRNA i and miRNA j as follows:

$${SM}^{2}\left(i,j\right)=\frac{\sum_{g\in {GS}_{i}}S\left(g,{GS}_{j}\right)+\sum_{g\in {GS}_{j}}S\left(g,{GS}_{i}\right)}{\left|{GS}_{i}\right|+\left|{GS}_{j}\right|}\;,$$ (7)

where ${GS}_{i}$ and ${GS}_{j}$ are gene sets associated with miRNA i and j , respectively. Similar to the miRNA sequence similarity network construction, we use the calculated miRNA functional similarity score as the edge weight of the network.

In addition, for miRNAs and diseases, different kinds of similarity matrix views obtained from different data sources are considered as their initial attribute feature, which can be used to further learn their attribute representations.

#### 2.2.6 Heterogeneous networks

By integrating the MDA network, two miRNA similarity networks and two disease similarity networks, multiple miRNA-disease heterogeneous networks are constructed, as shown in Figure 1A. As mentioned above, in each heterogeneous network, the weights of the edges between two miRNA nodes and between two disease nodes are equal to the similarity scores between them, respectively, while the edge weight between a miRNA node and a disease node is determined by whether there is a known association between two nodes. Given the MDA matrix A and similarity matrices ${SD}^{1},\;{SD}^{2},\;{SM}^{1}$ and ${SM}^{2}$ , we define the adjacency matrices M of these heterogeneous networks as follows:

$$M^{1}=\left[\begin{array}{cc} {SM}^{1} & A\\ A^{T} & {SD}^{1} \end{array}\right]\in R^{\left(N_{m}+N_{d}\right)\times \left(N_{m}+N_{d}\right)}\;,$$

$$M^{2}=\left[\begin{array}{cc} {SM}^{1} & A\\ A^{T} & {SD}^{2} \end{array}\right]\in R^{\left(N_{m}+N_{d}\right)\times \left(N_{m}+N_{d}\right)}\;,$$

$$M^{3}=\left[\begin{array}{cc} {SM}^{2} & A\\ A^{T} & {SD}^{1} \end{array}\right]\in R^{\left(N_{m}+N_{d}\right)\times \left(N_{m}+N_{d}\right)}\;,$$

$$M^{4}=\left[\begin{array}{cc} {SM}^{2} & A\\ A^{T} & {SD}^{2} \end{array}\right]\in R^{\left(N_{m}+N_{d}\right)\times \left(N_{m}+N_{d}\right)}\;,$$

where $A^{T}$ denotes the transpose of A , and $M^{1},\;M^{2},\;M^{3}$ and $M^{4}$ respectively represent the matrix representations of four different heterogeneous network views, which reflect the relationship between miRNAs and diseases and the degree of similarity between nodes of the same type from the perspective of different information sources.

### 2.3 Multi-view topology representation learning

#### 2.3.1 Topology representations learning by graph convolutional network encoder

Graph convolutional network (GCN) is a powerful tool for learning node embeddings of graph-structured data, which has been proven both theoretically and practically (Zhou et al., 2020). GCN generates a low-dimensional and efficient representation of a node by aggregating the information of the neighbour nodes of the node in the graph and capturing the dependencies between the data. For an undirected graph, the layer-wise propagation rule of a multi-layer GCN can be expressed as follows:

$$X^{\left(l+1\right)}=\sigma \left(D^{-\frac{1}{2}}GD^{-\frac{1}{2}}X^{\left(l\right)}W^{\left(l\right)}\right)\;,$$ (8)

Where $X^{\left(l\right)}$ is the representations of the nodes in the l -th layer, σ denotes the nonlinear activation function, and $W^{\left(l\right)}$ is the learnable weight matrix that maps the features to the latent space. G is the adjacency matrix of the graph, and D is the diagonal degree matrix of G , $D_{ii}=\sum_{j}G_{ij}$ , where i, j=1, 2,⋯,N and N represents the number of nodes in the graph.

For the miRNA-disease heterogeneous network views constructed in the previous chapter, we use them as the input of the GCN encoder respectively, and then obtain different embeddings of miRNAs and diseases. Taking the use of GCN to encode the heterogeneous network $M^{1}$ as an example, we set $G=M^{1}$ , and then according to Eq. 8, the first layer of the GCN encoder can be defined as follows:

$$X_{md1}^{\left(1\right)}=\sigma \left(D_{md1}^{-\frac{1}{2}}M^{1}D_{md1}^{-\frac{1}{2}}X_{md1}^{\left(0\right)}W_{md1}^{\left(0\right)}\right)\;,$$ (9)

where $W_{dm1}^{\left(0\right)}\in R^{\left(N_{m}+N_{d}\right)\times f_{md}}$ is the weight matrix input to the hidden layer, $f_{md}$ represents the dimension of the embedding feature, and the initial embedding $X_{dm1}^{\left(0\right)}=M^{1}$ . According to Eq. 8, we can get the embedding of the heterogeneous network $M^{1}$ as follows:

$$X_{md1}=\left\{X_{md1}^{\left(1\right)},\;X_{md1}^{\left(2\right)},\cdots ,X_{md1}^{\left(L\right)}\right\}\;,$$ (10)

where the GCN has L layers to learn topology information of the $M^{1}$ heterogeneous network view.

Similarly, we can obtain the embedding $X_{mdi}$ of miRNA and disease according to the miRNA-disease heterogeneous network view $M^{i}$ in turn, where i=2, 3,⋯,n and n is the number of the heterogeneous network views. The obtained embeddings can be represented as:

$$X_{md2}=\left\{X_{md2}^{\left(1\right)},\;X_{md2}^{\left(2\right)},\cdots ,X_{md2}^{\left(L\right)}\right\}\;,$$ (11)

$$X_{md3}=\left\{X_{md3}^{\left(1\right)},\;X_{md3}^{\left(2\right)},\cdots ,X_{md3}^{\left(L\right)}\right\}\;,$$ (12)

⋮

$$X_{mdn}=\left\{X_{mdn}^{\left(1\right)},\;X_{mdn}^{\left(2\right)},\cdots ,X_{mdn}^{\left(L\right)}\right\}\;.$$ (13)

Furthermore, as shown in Figure 1B, we get topological representations of miRNAs and diseases from different perspectives according to the multi-layer GCN encoder and the next section will describe how to integrate these representations, which contain different structural information.

#### 2.3.2 Topology representations fusing by attention mechanism

The structural information of the input network captured by different GCN layers is different. For instance, the first layer captures the direct connection information between nodes, and by updating the embeddings layer by layer, multi-hop neighbour information can be captured by higher layer embeddings (He et al., 2020; Yu et al., 2021). In addition, the embeddings from different heterogeneous network views are not equally important to explore MDAs. Therefore, we design the attention strategy at the topology representation level to adaptively fuse multiple topology embeddings of miRNAs and diseases learned by GCN encoder. The multiple feature matrices of miRNAs and diseases from the heterogeneous network views are stacked to form a feature tensor $X_{md}\in R^{\left(N_{m}+N_{d}\right)\times f_{md}\times \left(n\times L\right)}$ . Given the feature tensor of $X_{md}$ , the attention weight $\beta_{a}$ is calculated as follows:

$$s_{a}^{i}=q_{a}^{T}\tanh\left({W_{a}X_{md}}^{i}+b_{a}\right)\;,$$ (14)

$$\beta_{a}^{i}=\frac{\exp\left(s_{a}^{i}\right)}{\sum_{j=1}^{n\times L}\exp\left(s_{a}^{j}\right)},$$ (15)

where $W_{a}$ , $b_{a}$ and $q_{a}$ denote the weight matrix, the bias vector and the topology representation attention vector, respectively. $s_{a}^{i}$ is the information score of the i -th topology representation. After obtaining the attention score at topology representation level, we could focus on more important features by combining the topology representations with attention, and the topological features $\left[\begin{array}{c} Z_{m}^{1}\\ Z_{d}^{1} \end{array}\right]\in R^{\left(N_{m}+N_{d}\right)\times f_{md}}$ after attention enhancement are expressed as follows:

$$\left[\begin{array}{c} Z_{\text{m}}^{1}\\ Z_{\text{d}}^{1} \end{array}\right]=\sum_{i=1}^{n\times L}\beta_{a}^{i}X_{md}^{i}.$$ (16)

### 2.4 Multi-view attribute representations learning by convolutional neural network encoder

Convolutional neural network (CNN) can obtain the local message contained in the feature map through multiple convolution kernels, which helps us to use CNN to extract the deep attribute features of miRNAs and diseases from different information sources respectively. We take P different kinds of miRNA similarity matrix views as the initial attribute feature of miRNAs $SM=\left[{SM}^{1},\;{\;SM}^{2},\cdots ,\;{\;SM}^{P}\right]$ and regard Q different disease similarity matrix views as the initial attribute feature of disease $SD=\left[{SD}^{1},\;{\;SD}^{2},\cdots ,\;{\;SD}^{Q}\right]$ . Given the initial attribute feature SM of miRNAs nodes, the embedding of t -th output channel ${output}_{t}$ is expressed as:

$${output}_{t}=\overset{P}{\underset{i=1}{\sum }}{SM}^{i}⊗W_{m}^{t}+b_{m}^{t},$$ (17)

where $W_{m}^{t}\in R^{N_{m}\times 1}$ represents the t -th convolution filter, $b_{m}^{t}\in R^{N_{m}\times 1}$ denotes the bias vector, and ⊗ is the convolution operator. The final miRNA attribute representation $Z_{m}^{2}\in R^{N_{m}\times f_{channel}}$ can be got by stacking the output embeddings of multiple channels, where $f_{channel}$ is the number of the output channels. Similarly, as shown in Figure 1D, we can extract the disease attribute representation $Z_{d}^{2}\in R^{N_{d}\times f_{channel}}$ by leveraging the CNN encoder to the initial attribute feature SD .

In order to make full use of the information from different data sources, we combine the topological features from multiple miRNA-disease heterogeneous networks learned by GCN encoder and the attribute features from multiple similarity matrices learned by CNN encoder as the final embeddings, which is expressed as follows:

$$Z_{m}=Z_{m}^{1}\;⊕\;Z_{m}^{2},$$ (18)

$$Z_{d}=Z_{d}^{1}\;⊕\;Z_{d}^{2},$$ (19)

where ⊕ represents the concatenation operation, and $Z_{m}$ and $Z_{d}$ are respectively the final embeddings of miRNA nodes and disease nodes.

#### 2.4.1 The reconstruction of miRNA-disease associations and optimization

Although the inner product of node embeddings is often used to predict relationship probabilities between nodes, it is limited in capturing complex associations between nodes. Here, we reconstruct the associations between miRNAs and diseases by introducing a bilinear decoder. Based on the obtained embedding matrices $Z_{m}\in R^{N_{m}\times \left(f_{md}+f_{channel}\right)}$ for miRNAs and $Z_{d}\in R^{N_{d}\times \left(f_{md}+f_{channel}\right)}$ for diseases, the prediction scores between miRNAs and diseases are calculated as follows:

$$A^{\prime }=sigmoid\left(Z_{m}W_{b}Z_{d}^{T}\right)\;,$$ (20)

where $W_{b}$ denotes a learnable matrix, and $A_{i,j}^{\prime }$ is the prediction probability that miRNA i and disease j are associated. The higher the predicted score between miRNA i and disease j , the more likely that miRNA i is correlated with disease j .

In this study, we train MVIFMDA to learn the parameters of the model by utilizing the following cross-entropy loss L :

$$L=-\left(\sum_{\left(i,j\right)\in y^{+}}\log A_{i,j}^{\prime }+\sum_{\left(i,j\right)\in y^{-}}\log\left(1-A_{i,j}^{\prime }\right)\right)\;,$$ (21)

where $y^{+}$ and $y^{-}$ represents the set of positive samples and the set of negative samples, respectively. The known MDA pairs are regarded as the positive samples and the other unobserved pairs are considered as negative samples. In addition, we leverage the Adam optimizer (Kingma and Ba, 2014) to minimize the loss function and train the model end-to-end by a back propagation algorithm.

## 3 Results

### 3.1 Experiment settings

In this study, 5-CV is adopted to evaluate the performance of MVIFMDA for identifying candidate disease-associated miRNAs. For 5-CV, all known MDAs (also named positive samples) are randomly divided into five equal parts, and each part in turn is utilized for testing while the remaining is adopted for training. In each fold, the miRNA-disease heterogeneous networks are updated based on new known associations, where the known associations for testing are treated as unobserved associations. MDA prediction can be viewed as a classification task, therefore, several common classification metrics are used to evaluate the prediction performance of MVIFMDA and baseline models, including area under receiver operating characteristic (ROC) curve (AUC), area under the precision-recall (PR) curve (AUPR), accuracy, precision, recall, specificity, and precision rate and recall rate within the top k . In addition, we set k={5%, 10%} in the study and repeat the experiments 10 times to get the average of these metrics.

In MVIFMDA, there are several hyperparameters to adjust, such as the number of GCN layer L , topology embedding dimension for miRNAs and diseases $f_{md}$ , attribute embedding dimension $f_{channel}$ and learning rate lr . By adjusting the parameters empirically, we set the parameter L=2 , $f_{md}=256$ , $f_{channel}=256$ , lr=0.001 for the MVIFMDA model. In addition, we take the optimal values given by the authors as the parameters of the baseline models.

### 3.2 Ablation experiments

In the study, we combine GCN encoder and CNN encoder to enhance the feature embeddings of miRNAs and diseases. In order to validate the effectiveness of the main components in MVIFMDA, we designed two variants of MVIFMDA (MVIFMDA-noTR, MVIFMDA-noAR) for the ablation study. MVIFMDA-noTR removes the topology representations of miRNAs and diseases based on the GCN encoder. MVIFMDA-noAR only adopts the topology features without using the attribute representations based on the CNN encoder. The experimental results of MVIFMDA and two variants are shown in Table 1. The results demonstrate that MVIFMDA outperforms the other two variant models on all evaluation metrics. It means that the topological representations obtained by the GCN encoder and the attribute representations learned by the CNN encoder can play a complementary role and the combination of the two can more effectively learn the multi-view information of miRNA and disease nodes from different information sources. For MVIFMDA-noTR and MVIFMDA-noAR, the performance of MVIFMDA-noAR is better, i.e., the topological information extracted from the heterogeneous network views is very useful and the topological level attention mechanism effectively integrates the different structural information. In conclusion, the combination of topological representations from multiple heterogeneous network views learned by the GCN encoder and attribute representations from multiple similarity views learned by the CNN encoder makes our proposed model perform better.

**TABLE 1:** Results of our model and its variant models.

Metrics ( mean±std )	MVIFMDA-noTR	MVIFMDA-noAR	MVIFMDA
AUC	0.9063±0.0350	0.9371±0.0003	0.9396±0.0004
AUPR	0.2024±0.0123	0.2591±0.0009	0.2696±0.0009
accuracy	0.9931±0.0002	0.9935±0.0001	0.9936±0.0002
precision	0.2886±0.0171	0.3420±0.0073	0.3494±0.0118
recall	0.2515±0.0173	0.2962±0.0066	0.2982±0.0080
F1	0.2674±0.0163	0.3162±0.0019	0.3202±0.0006
precision@5%	0.0632±0.0037	0.0696±0.0002	0.0701±0.0002
precision@10%	0.0382±0.0022	0.0414±0.0001	0.0416±0.00003
recall @5%	0.6404±0.0374	0.7045±0.0018	0.7097±0.0018
recall @10%	0.7576±0.0433	0.8214±0.0010	0.8261±0.0006

The bold values indicate the best values in rows.

### 3.3 Comparison with other methods

To demonstrate the performance of MVIFMDA in identifying potential disease-related miRNAs, we compared it with six state-of-the-art approaches that were developed for MDA prediction, including MDHGI (Chen et al., 2018b), ABMDA (Zhao et al., 2019), NIMGSA (Jin et al., 2022), NIMCGCN (Li et al., 2020), DANE-MDA (Ji et al., 2021), MMGCN (Tang et al., 2021).

**FIGURE 2:**
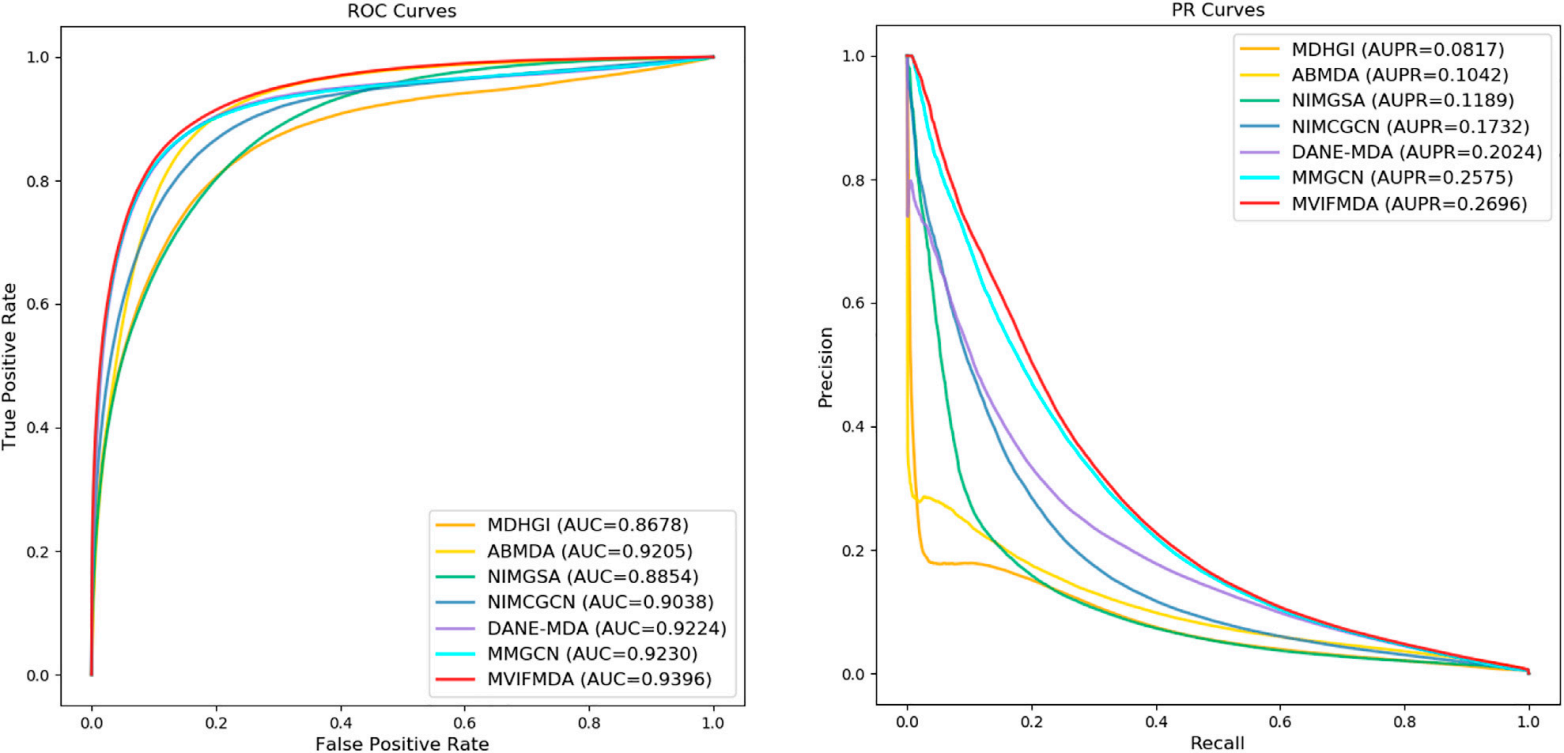
ROC curves and PR curves of MVIFMDA with all comparison methods.

For a fair comparison, all models are evaluated using 5-CV. Figure 2 and Table 2 show that except that the recall of MVIFMDA is slightly lower than that of MMGCN, all other metrics are significantly higher than the comparison methods, whereas compared to MMGCN, the AUC and AUPR are improved by 1.8 and 4.7%, respectively. One of the possible reasons is that MVIFMDA is able to enhance the representation of nodes by combining topological representations from different heterogeneous network views and attribute representations from different similarity views, which further shows that the design of our model is sound. Compared with MDHGI, ABMDA, NIMGSA, and NIMCGCN, MVIFMDA builds multiple heterogeneous networks with different similarities and learns topological representations from these heterogeneous networks respectively, and uses CNNs to learn high-level features from multiple similarity matrices, which replaces simply combining the multiple similarities into one like the compared methods. Although DANE-MDA considers both the attribute information and topology information, its performance is not as good as MVIFMDA. This may be because of that DANE-MDA simply uses miRNA sequence similarity and disease semantic similarity to obtain node embeddings of miRNAs and diseases, while MVIFMDA learns topological and attribute information from two different similarities more efficiently. Furthermore, although MMGCN uses a multichannel attention mechanism to capture more important topological features from different similarity network views, its performance is also not as good as that of MVIFMDA in addition to the recall, probably because MVIFMDA is capable of combining topological representations learned by GCN and attribute representations learned by CNN to more effectively capture information in multi-view networks. In addition, we further evaluate the performance of MVIFMDA and the comparison methods using the paired *t*-test based on 10 runs of 5-CV. Table 3 shows that MVIFMDA is significantly preferred to other computational methods in terms of AUC and AUPR ( P<0.05 ).

**TABLE 2:** The performance of MVIFMDA with all comparison methods.

Metrics ( mean±std )	MDHGI	ABMDA	NIMGSA	NIMCGCN	DANE-MDA	MMGCN	MVIFMDA
AUC	0.8678±0.0008	0.9205±0.0036	0.8854±0.0022	0.9038±0.0064	0.9224±0.0006	0.9230±0.0023	0.9396±0.0004
AUPR	0.0817±0.0012	0.1042±0.0154	0.1189±0.0089	0.1732±0.0174	0.2024±0.0030	0.2575±0.0008	0.2696±0.0009
accuracy	0.9894±0.0004	0.9851±0.0088	0.9908±0.0008	0.9928±0.0007	0.9933±0.0008	0.9934±0.0001	0.9936±0.0002
precision	0.1428±0.0101	0.0690±0.0448	0.1680±0.0232	0.2569±0.0292	0.1559±0.0819	0.3338±0.0081	0.3494±0.0118
recall	0.2200±0.0135	0.1416±0.0932	0.1891±0.0156	0.2146±0.0203	0.1683±0.0861	0.2986±0.0067	0.2982±0.0080
F1	0.1719±0.0109	0.0751±0.0452	0.1741±0.0161	0.2305±0.0202	0.1603±0.0826	0.3141±0.0007	0.3202±0.0006
precision@5%	0.0504±0.0003	0.0553±0.0028	0.0500±0.0008	0.0589±0.0016	0.0689±0.0002	0.0695±0.0001	0.0701±0.0002
precision@10%	0.0329±0.0001	0.0382±0.0009	0.0323±0.0004	0.0371±0.0007	0.0410±0.0001	0.0412±0.0001	0.0416±0.00003
recall @5%	0.5102±0.0027	0.5607±0.0279	0.5062±0.0078	0.5961±0.0165	0.6979±0.0018	0.7038±0.0010	0.7097±0.0018
recall @10%	0.6525±0.0027	0.7579±0.0171	0.6412±0.0088	0.7370±0.0136	0.8150±0.0018	0.8179±0.0016	0.8261±0.0006

The bold values indicate the best values in rows.

**TABLE 3:** The statistical results by paired *t*-test for MVIFMDA and all comparison methods.

MVIFMDA versus	MDHGI	ABMDA	NIMGSA	NIMCGCN	DANE-MDA	MMGCN
P -vaule of AUC	6.7944e-18	1.1456e-07	6.5493e-14	3.5915e-08	2.0171e-13	9.7205e-09
P -vaule of AUPR	2.0333e-19	1.3929e-10	2.5665e-12	4.7847e-08	1.7074e-13	2.6278e-10

### 3.4 Case studies: Colonic neoplasms, esophageal neoplasms and lymphoma

To further demonstrate the reliability of the MVIFMDA model in real cases, we construct case studies for colonic neoplasms, esophageal neoplasms and lymphoma. All known MDAs are used as positive samples to train the model, then this trained model is used to predict the probability scores of all unknown relationship pairs. For each disease, the predicted scores are sorted in descending order.

The top-rank 20 miRNAs associated with each disease are shown in Tables 4–6, where we use dbDEMC 3.0 (Yang et al., 2017) to validate the candidate MDAs. Colon tumours are the third leading cause of cancer-related deaths in the United States (Siegel et al., 2016). As shown in Table 4, among the top-rank 20 disease-related candidate miRNAs, 18 are identified by dbDEMC, which suggests that these miRNAs are associated with colonic neoplasms. Overexpression of hsa-miR-122 increases the sensitivity of fluorouracil (5-FU)-resistant colon cancer cells to 5-FU through PKM2 downregulation (He et al., 2014). High levels of hsa-miR-122-5p in plasma could suggest liver metastases from colorectal cancer and correlate with poorer recurrence-free survival and overall survival times (Maierthaler et al., 2017; Sun et al., 2020). Colorectal cancer is a collective term for colon cancer and rectal cancer, implying that hsa-miR-122-5p may be associated with the survival prognosis of colon cancer patients. High levels of miR-196a in colorectal cancer can actuate the Akt signaling pathway and accelerate cancer cell metastasis and infiltration (Schimanski et al., 2009; Wang et al., 2010b). Furthermore, it was mentioned in (Ge et al., 2014) that miR-196a in colorectal cancer displays an association with aggressive disease and a detrimental effect on therapeutic outcomes. Esophageal tumours are the major malignant tumours of the digestive system, with the sixth and fourth highest incidence and mortality rates, respectively, among all malignancies. Lymphoma, meanwhile, is a malignant tumour of the lymphatic hematopoietic system, the incidence of which is increasing annually. From Tables 5, 6, it can be seen that the top-rank 20 candidate miRNAs predicted by the MVIFMDA model with regard to esophageal neoplasms and lymphomas can all be confirmed by the dbDEMC dataset. In summary, the case studies further show that our model is effective in inferring new disease-related miRNAs.

**TABLE 4:** Top 20 miRNA candidates related to colonic neoplasms.

Rank	miRNA	Evidence	Rank	miRNA	Evidence
1	hsa-mir-122	Unconfirmed	11	hsa-mir-100	dbDEMC
2	hsa-mir-146b	dbDEMC	12	hsa-mir-34b	dbDEMC
3	hsa-mir-182	dbDEMC	13	hsa-mir-149	dbDEMC
4	hsa-mir-214	dbDEMC	14	hsa-mir-342	dbDEMC
5	hsa-mir-29c	dbDEMC	15	hsa-mir-26b	dbDEMC
6	hsa-mir-27b	dbDEMC	16	hsa-mir-196a-2	Unconfirmed
7	hsa-mir-206	dbDEMC	17	hsa-mir-193b	dbDEMC
8	hsa-mir-183	dbDEMC	18	hsa-mir-99a	dbDEMC
9	hsa-mir-34c	dbDEMC	19	hsa-mir-29b-2	dbDEMC
10	hsa-mir-144	dbDEMC	20	hsa-mir-494	dbDEMC

**TABLE 5:** Top 20 miRNA candidates related to esophageal neoplasms.

Rank	miRNA	Evidence	Rank	miRNA	Evidence
1	hsa-mir-17	dbDEMC	11	hsa-mir-23a	dbDEMC
2	hsa-mir-29a	dbDEMC	12	hsa-mir-125a	dbDEMC
3	hsa-mir-222	dbDEMC	13	hsa-mir-15b	dbDEMC
4	hsa-mir-142	dbDEMC	14	hsa-mir-206	dbDEMC
5	hsa-mir-30a	dbDEMC	15	hsa-mir-125b-1	dbDEMC
6	hsa-mir-132	dbDEMC	16	hsa-mir-23b	dbDEMC
7	hsa-mir-18a	dbDEMC	17	hsa-mir-16-1	dbDEMC
8	hsa-mir-200b	dbDEMC	18	hsa-let-7d	dbDEMC
9	hsa-mir-182	dbDEMC	19	hsa-mir-125b-2	dbDEMC
10	hsa-mir-19b-1	dbDEMC	20	hsa-mir-107	dbDEMC

**TABLE 6:** Top 20 miRNA candidates related to lymphoma.

Rank	miRNA	Evidence	Rank	miRNA	Evidence
1	hsa-mir-34a	dbDEMC	11	hsa-mir-132	dbDEMC
2	hsa-mir-223	dbDEMC	12	hsa-mir-23a	dbDEMC
3	hsa-mir-145	dbDEMC	13	hsa-mir-182	dbDEMC
4	hsa-mir-29a	dbDEMC	14	hsa-mir-192	dbDEMC
5	hsa-mir-30a	dbDEMC	15	hsa-mir-214	dbDEMC
6	hsa-let-7b	dbDEMC	16	hsa-mir-15b	dbDEMC
7	hsa-mir-195	dbDEMC	17	hsa-mir-183	dbDEMC
8	hsa-mir-106b	dbDEMC	18	hsa-let-7c	dbDEMC
9	hsa-mir-146b	dbDEMC	19	hsa-mir-130a	dbDEMC
10	hsa-mir-27a	dbDEMC	20	hsa-mir-205	dbDEMC

### 3.5 Prediction of novel diseases

To show the predictive performance of MVIFMDA for new diseases without known relevant miRNAs, we construct another case study for novel diseases in this experiment. When predicting miRNAs relevant to a new disease, we use known relationship pairs other than those associated with the specific disease as positive samples to train the MVIFMDA model and then explore the relationship probabilities between the specific disease and all miRNAs. Based on the descending ranked prediction scores, we use HMDD v3.2 to verify these top-rank 20 candidate MDA pairs.

Breast cancer is the most common cancer worldwide, and miRNAs are considered as new diagnostic and prognostic markers for it. Therefore, here we predict miRNAs associated with breast neoplasms by employing the MVIFMDA model, and as shown in Table 7, 20 of the top 20 miRNAs are validated by the HMDD dataset, which show that our model is good in identifying miRNAs associated with novel diseases.

**TABLE 7:** Top 20 miRNA candidates related to breast neoplasms. The miRNAs associated with breast neoplasms are deleted before training the MVIFMDA model.

Rank	miRNA	Evidence	Rank	miRNA	Evidence
1	hsa-mir-21	HMDD	11	hsa-mir-210	HMDD
2	hsa-mir-155	HMDD	12	hsa-mir-221	HMDD
3	hsa-mir-146a	HMDD	13	hsa-mir-20a	HMDD
4	hsa-mir-126	HMDD	14	hsa-mir-19a	HMDD
5	hsa-mir-150	HMDD	15	hsa-mir-146b	HMDD
6	hsa-mir-223	HMDD	16	hsa-mir-142	HMDD
7	hsa-mir-34a	HMDD	17	hsa-mir-143	HMDD
8	hsa-mir-17	HMDD	18	hsa-mir-122	HMDD
9	hsa-mir-145	HMDD	19	hsa-mir-222	HMDD
10	hsa-mir-29a	HMDD	20	hsa-mir-22	HMDD

## 4 Conclusion

In this study, we propose a new end-to-end model called MVIFMDA to predict potential MDAs. This model captures topological features in multiple heterogeneous network views by GCN encoder, then adaptively fuses different topological features using an attention mechanism, furthermore employs CNN encoder to extract attribute features from different similarity views of miRNAs and diseases, respectively, finally its prediction performance is improved by combining topological and attribute features. The comparison with six advanced methods for identifying new MDAs and the case studies indicate that MVIFMDA has excellent predictive performance and can perform well in practical applications.

Although MVIFMDA has shown good predictive performance, it still has some issues that need further investigation. First, we use CNN to learn the attribute representations of miRNA and disease node levels, whether the attribute embeddings of miRNA and disease node pairs levels can improve the prediction performance of MDAs needs to be further studied. Second, we use only two similarities of both miRNAs and diseases, and more relevant evidence of miRNA and disease should be used to construct the similarity networks, such as the interaction relationships between miRNAs and lncRNAs and the association relationships between lncRNAs and diseases. In addition, though we consider using gene-related information to calculate the similarity of diseases and miRNAs, a multi-layer network among genes, miRNAs and diseases is not directly constructed to explore the miRNAs correlated with diseases. Therefore, it is still worthwhile to continue investigating how to effectively utilize the information from multiple data sources.

## Data Availability

The original contributions presented in the study are included in the article/Supplementary Material, further inquiries can be directed to the corresponding author.
